# How to measure humeral medialization and upward migration after shoulder hemiarthroplasty

**DOI:** 10.1016/j.jseint.2026.101655

**Published:** 2026-02-10

**Authors:** Philipp Schippers, Caroline Cointat, Marc-Olivier Gauci, Yann Pelletier, Jean-Luc Raynier, Pascal Boileau

**Affiliations:** a Department of Orthopedics and Traumatology, University Medical Center of the Johannes Gutenberg-University, Mainz, Germany; b Pôle Santé Saint Jean, Cagnes-sur-Mer, France; c IULS (Institut Universitaire Locomoteur & du Sport), Hôpital Pasteur 2, Nice, France; d ICR - Institut de Chirurgie Réparatrice - Locomoteur & Sport, Clinique Kantys Centre, Nice, France

**Keywords:** Glenohumeral osteoarthritis, Hemiarthroplasty, Glenoid erosion, Humeral medialization, Humeral upward migration, Pyrolytic carbon

## Abstract

**Background:**

The 2 main complications after hemiarthroplasty (HA) are glenoid erosion, resulting in humeral medialization, and rotator cuff insufficiency, resulting in upward migration of the humerus. Currently, qualitative assessments of glenoid erosion, like the radiographic Sperling classification, are primarily used. Hence, we set out to present and validate a novel radiographic method for quantitatively assessing humeral medialization and upward migration following HA implantation.

**Methods:**

The immediate postoperative and last follow-up anteroposterior (AP) radiographs from 64 consecutive patients who underwent a pyrocarbon HA for symptomatic osteoarthritis were used to validate the new method. The new method is based on an orthonormal frame drawn upon AP radiographs in neutral rotation. The X-axis corresponds to the supraspinatus fossa line, and the Y-axis is the orthogonal line tangent to the most lateral border of the acromion. Humeral medialization and upward migration were measured and normalized with the humerus shaft diameter to avoid scale errors. Four independent observers applied the new measurements and determined the Sperling classification for glenoid erosion on all radiographs. Measurement reliability was evaluated using intraclass correlation (ICC) and Fleiss' Kappa coefficients.

**Results:**

Interobserver reliability was good to excellent: ICC for humeral medialization = 0.96 (confidence interval: 0.93-0.97; *P* < .00) and ICC for upward migration = 0.88 (confidence interval: 0.83-0.93; *P* < .001). A moderate positive correlation (r = 0.54, *P* < .0001) was observed between humeral medialization and the progression of glenoid erosion as classified by Sperling. After a mean follow-up of 31 months (range: 24-50 months), humeral medialization averaged 18% (±17) of the shaft diameters, while humeral upward migration averaged 13% (±14) of the shaft diameters. Normalized per month, medialization was 0.57% (±0.55) and upward migration was 0.44% (±0.51) of the shaft diameter. Finally, 39% of patients showed almost no glenoid erosion, and 44% showed almost no upward migration.

**Conclusions:**

The new radiologic measurement method, based upon sequential postoperative AP radiographs, can be reliably used to measure humeral medialization and upward migration of HA overtime. Despite a low elastic modulus, pyrocarbon HA still leads to significant glenoid erosion in about 60% of the patients. Here proposed measurement method can help precisely quantify the speed of medialization, thereby enabling the identification of risk factors in patients with pronounced medialization in future studies.

The 2 main complications after hemiarthroplasty (HA) are glenoid erosion, resulting in humeral medialization, and rotator cuff insufficiency, resulting in upward migration of the humerus.^19^ Pyrolytic carbon is a relatively new material that was initially developed for nuclear reactors and the space industry and later introduced into the medical field, where it is marketed under the name of “PyroCarbon” (PyC). PyC has an elastic modulus that resembles bone more than conventional metal and is thus said to cause less bone or cartilage erosions.^20^

Pyrocarbon hemiarthroplasty (HA-PyC) proved an effective treatment option in young, arthritic patients with an intact rotator cuff after exhaustion of conservative treatment options.^3^ Several studies have reported promising short- and mid-term outcomes.^2^^,^^3^^,^^6^^,^^11^^,^^13^^,^^17^^,^^26^ In contrast, other studies report revision rates of up to 21.7% in patients undergoing HA-PyC before 60 years.^7^

Evaluating glenoid erosion and humeral upward migration is of paramount importance. The Sperling classification can assess glenoid erosion; however, this classification remains qualitative.^21^^,^^24^ Thus, there is a need to quantitatively assess glenoid erosion.^19^ Hence, the goal of the present study was to develop and validate a novel radiographic method to measure humeral medialization and upward migration after HA. We hypothesized that true anteroposterior (AP) radiographs could be reliably used to measure medialization and upward migration of HA overtime. We also hypothesized that despite its favorable elastic modulus, HA-PyC still exhibits medialization and upward migration.

## Methods

### Study design

This multicenter, retrospective study enrolled anonymized radiographs of all consecutive patients who underwent HA-PyC for symptomatic glenohumeral osteoarthritis in 5 dedicated French centers for shoulder surgery between 2013 and 2017. Radiographs of patients aged 60 or older with at least 2 years of follow-up were included. Exclusion criteria were previous shoulder arthroplasty and previous surgery for rotator cuff tears. Sixty-four patients with a mean follow-up of 31.1 months (range: 24-50) were included. The cohort consisted of 45 right and 19 left shoulders.

### Surgical procedure

All surgeries (arthroplasties) were performed through a deltopectoral approach, under general and local anesthesia. All cases were planned preoperatively using Blueprint planning software (Stryker, Kalamazoo, MI, USA) to determine the ideal size of the prosthetic head and stem. The Aequalis Ascend FlexTM stem (Wright Medical, Memphis, TN, USA) was implanted in all patients. The diameter of the pyrocarbon head varied between 39 and 50 mm, with care not to oversize the head.

### New measurement methods and radiographic assessment

**Figure 1:**
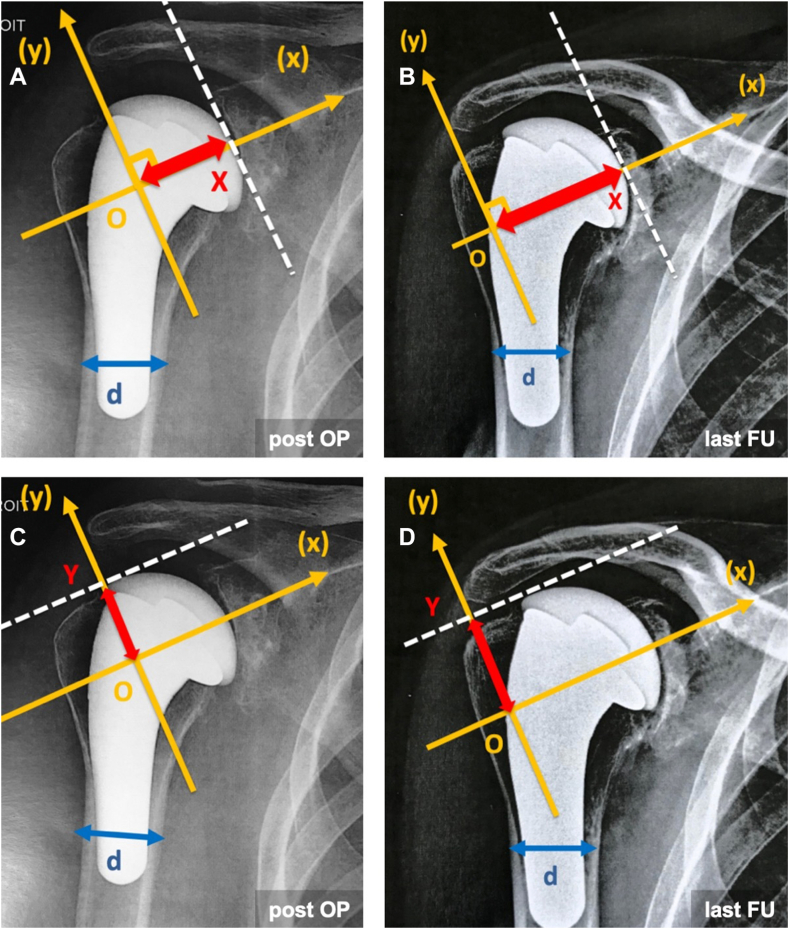
Measurements of humeral medialization and humeral upward migration - (x) is the axis of the supraspinatus fossa. (y) is the orthogonal line to (x) tangent to the lateral border of the acromion. Point O: intersection of (x) and (y). Point X: intersection of (x) and the orthogonal line to (x) tangent to the medial border of the prosthetic humeral head. Point Y: intersection of (y) and the orthogonal line to (y) tangent to the upper part of the humeral head. d: humeral shaft diameter from lateral to medial cortex at the inflexion point of the calcar. To overcome the problem of scaling, OX and OY measurements have been normalized to d. Medialization: ΔOX/d = OX(last FU)/d(last FU) – OX(post-op)/d(post-op). Upward migration: ΔOY/d = OY(last FU)/d(last FU) – OY(post-op)/d(post-op). (**A**) Measurement OX (post-op) on the immediate post-operative AP X-rays. (**B**) Measurement OX (last FU) on the last FU AP X-rays. (**C**) Measurement OY (post-op) on the immediate post-operative AP X-rays. (**D**) Measurement OY (last FU) on the last FU AP X-rays. *AP*, anteroposterior; *FU,* follow-up.

A new method for measuring humeral medialization and upward migration was developed using an orthonormal frame drawn upon AP radiographs in neutral rotation. Only true AP (“Grashey's view”) radiographs compliant with the following parameters were accepted: (1) neutral rotation of the arm, (2) superimposition of the anterior and posterior glenoid rim, (3) visualization of glenohumeral space, and (4) foreshortening of the coracoid process but not of the scapular body. The X-axis corresponds to the supraspinatus fossa line, and the Y-axis is the orthogonal line tangent to the most lateral border of the acromion. The X- and Y-axis intersection defines the point “0.” Next, a line orthogonal to the X-axis, tangent to the most medial part of the prosthesis' head, was drawn. The intersection represents the point “X.” Likewise, a line orthogonal to the Y-axis, tangent to the superior part of the prosthesis' head, was drawn. The intersection represents the point “Y” (Fig. 1). Medialization of the prosthesis (ie, glenoid erosion) was defined as an increase of the distance OX on successive AP radiographs in neutral rotation, and upward migration of the prosthesis was defined as an increase of the distance OY on successive AP radiographs in neutral rotation. To overcome magnification errors, medialization and upward migration were measured in relation to the shaft diameter (d) at the inflexion point of the calcar.

**Figure 2:**
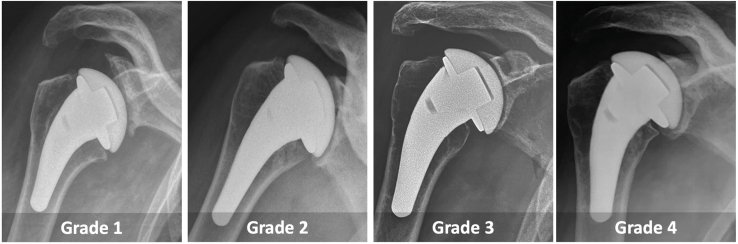
Sperling classification glenoid erosion was graded as follows: Grade 1: none; Grade 2: mild (erosion into subchondral bone); Grade 3: moderate (medialization beyond the subchondral bone with hemispheric deformation); Grade 4: severe (complete deformation/destruction of the glenoid or hemispheric deformation to/beyond the base of the coracoid).

Four observers (3 fellowship-trained shoulder surgeons and 1 radiologist) measured medialization and upward migration, according to the method described above, on immediate and last postoperative AP radiographs in neutral rotation. Glenoid erosion was also assessed according to the qualitative method proposed by Sperling et al^24^ (Fig. 2).

### Statistical analysis

Numerical outcomes were described as means with standard deviation. Grades of the Sperling classification were considered as ranked and thus ordinal. The Wilcoxon test was used to compare paired, nonparametric results. *P* values below 0.05 were considered statistically significant. Interobserver reliability was calculated between the 4 observers to assess the Sperling classification and calculate the medialization and the upward migration. For nominal results like the medialization and the upward migration, intraclass correlation (ICC) coefficients were calculated and interpreted according to Koo and Li,^15^ as shown in Table I. For ordinal results like the Sperling classification, Fleiss' Kappa coefficients were calculated, and results were interpreted according to Landis and Koch,^16^ as shown in Table I.

**Table I:** Interpretations for intraclass correlation and Fleiss' Kappa coefficients.

ICC	Interpretation	Fleiss' Kappa	Interpretation
>0.90	Excellent	>0.81	Almost perfect
>0.75	Good	>0.61	Substantial
>0.50	Moderate	>0.41	Moderate
≤0.50	Poor	>0.21	Fair
		>0.01	Slight

*ICC*, intraclass correlation.

For correlation analyses, Spearman's correlation coefficients were calculated, and results were interpreted according to Dancey and Reidy,^1^ as shown in Table II.

**Table II:** Interpretations for Spearman's correlation coefficients.

Spearman's r	Interpretation
≥0.7	Strong
≥0.4	Moderate
≥0.1	Weak
0	Zero

Statistical analyses were performed using SPSS 26 (IBM, Armonk, NY, USA) and GraphPad PRISM 10 (GraphPad Software, LLC, San Diego, CA, USA).

## Results

### Measurement reliability

The new method to quantitatively measure humeral medialization and upward migration on true AP radiographs after HA-PyC was introduced. The 4 observers independently determined humeral medialization (Delta-0X1/d–Delta-0X0/d) and upward migration (Delta-0Y1/d–Delta-0Y0/d) on the same radiographs. Medialization and upward migration were measured in relation to the shaft diameter (d). Interobserver reliability of the measurements was good to excellent (Table III).

**Table III:** Good to excellent interobserver reliability for humeral medialization and humeral upward migration.

	Humeral medialization	Humeral upward migration
Interobserver reliability (intraclass correlation)	**ICC_3,k_ = 0.96** CI = 0.93 – 0.97, *P* < .001	**ICC_3,k_ = 0.88** CI = 0.83 – 0.93, *P* < .001

*CI*, confidence interval; *ICC*, intraclass correlation.

There was good interobserver reliability for humeral medialization (Delta-OX1/d – Delta-OX0/d) and humeral upward migrations (Delta-0Y_1_/d – Delta-0Y_0_/d). Bold values indicate significant at *P* < .05.

The 4 observers independently determined the Sperling classification on the immediate postoperative and the last follow-up AP radiographs of 64 patients. There was only a slight interobserver reliability (Table IV).

**Table IV:** Slight interobserver reliability for the Sperling classification.

	Post-operative	Last follow-up
Interobserver reliability (Fleiss' Kappa)	**k = 0.16** CI = 0.09 – 0.22; *P* < .001	**k = 0.19** CI = 0.12 – 0.25; *P* < .001

*CI*, confidence interval.

There was only a slight interobserver reliability for the Sperling classification when determined between 4 observers on post-operative and on the last follow-up radiographs. Bold values indicate significant at *P* < .05.

### Medialization and upward migration

**Figure 3:**
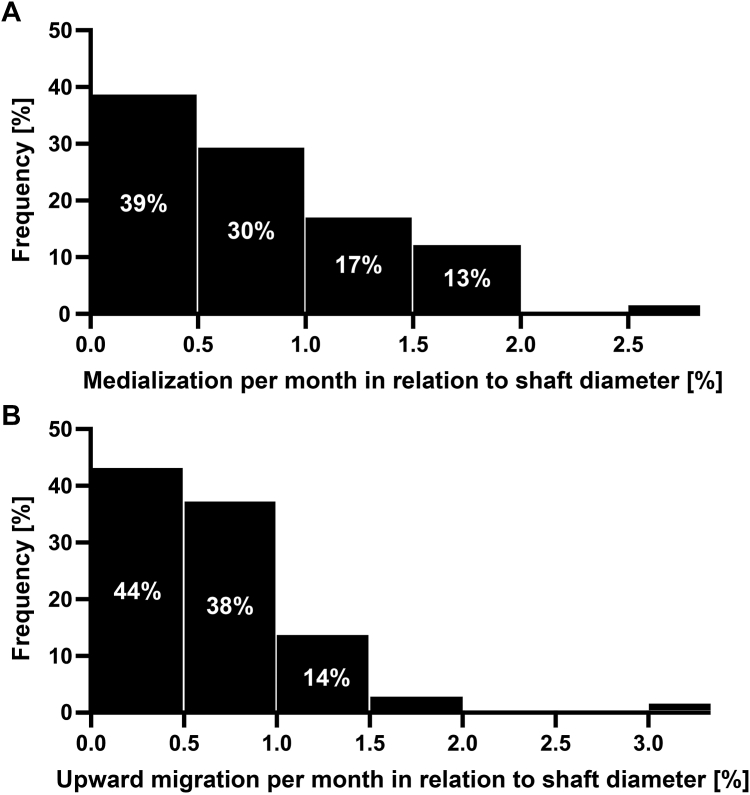
Frequency distribution of humeral medialization (**A**) and upward migration (**B**) per month in relation to shaft diameter. (**A**) 39% of patients showed almost no medialization per month, while 30% (17 + 13) showed a monthly medialization of 1-2% of the shaft diameter. (**B**) 44% of patients showed almost no upward migration, while 17% (14 + 3) showed a monthly Upward Migration of 1-2% of the shaft diameter. *FU,* follow-up.

Humeral medialization averaged 18% (±17%) of the shaft diameter, while upward migration averaged 13% (±14%) of the shaft diameter. To compensate for the different follow-up periods ranging from 24 to 50 months, medialization and upward migration were also normalized to the individual follow-up periods and thus calculated per month. Humeral medialization per month averaged 0.57% (±0.55%) of the shaft diameter. Upward migration per month averaged 0.44% (±0.51%) of the shaft diameter (Table V). Fig. 3 shows the distribution of the rate of humeral medialization and upward migration per month. While 39% of patients showed almost no medialization, 30% (17% + 13%) of patients experienced medialization of 1-2% of the shaft diameter per month (Fig. 3*A*). Likewise, 44% of patients showed almost no upward migration, yet in 17% (14% + 3%) of patients, a monthly upward migration of 1-2% of the shaft diameter was recorded (Fig. 3*B*).

**Table V:** Velocity of humeral medialization and humeral upward migration.

	Humeral medialization	Humeral upward migration
Mean (±SD) of shaft diameter		
Total/at last follow-up	**18%** (±17)	**13%** (±0.14)
Per month	**0.57%** (±0.55)	**0.44%** (±0.51)

SD, standard deviation.

Medialization and upward migration are shown on average at last follow-up and normalized to the individual follow-up periods. Bold values indicate significant at *P* < .05.

### Glenoid erosion according to Sperling

**Figure 4:**
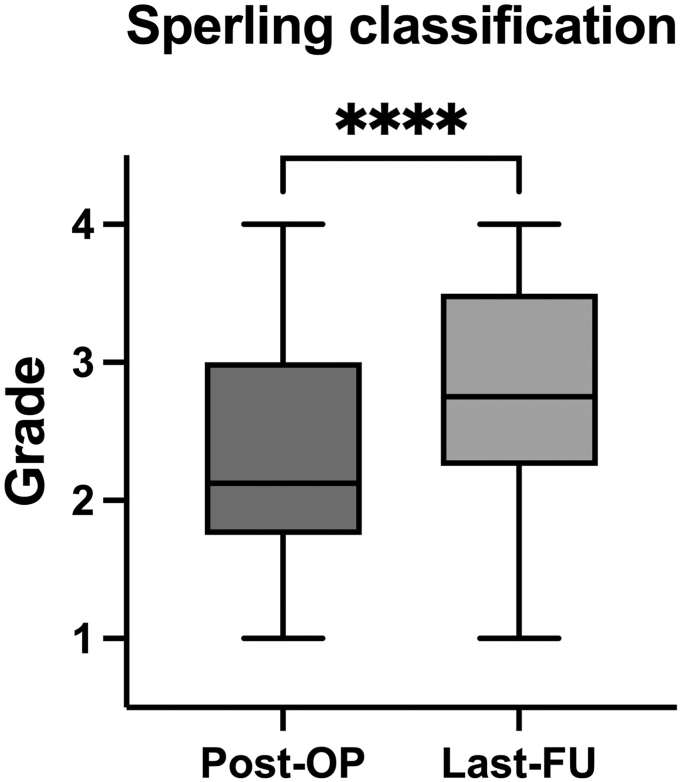
Increase of glenoid erosion according to Sperling classification. The Sperling classification was graded by 4 observers on immediate post-op and the last follow-up radiographs. Since the different grades of the Sperling classification can be ranked, grades can be considered ordinal and mean results were calculated. The mean grade significantly increased (*P* < .0001, Wilcoxon test) from 2.3 (SD = 0.8) to 2.8 (SD = 0.8). *SD*, standard deviation.

Since the different grades of the Sperling classification can be ranked, grades can be considered ordinal, and mean results were calculated. According to the Sperling classification, glenoid erosion increased from a mean grade of 2.3 (±0.8) postoperatively to a mean grade of 2.8 (±0.8) at the last follow-up (*P* < .0001, Wilcoxon test) (Fig. 4).

### Correlations

**Figure 5:**
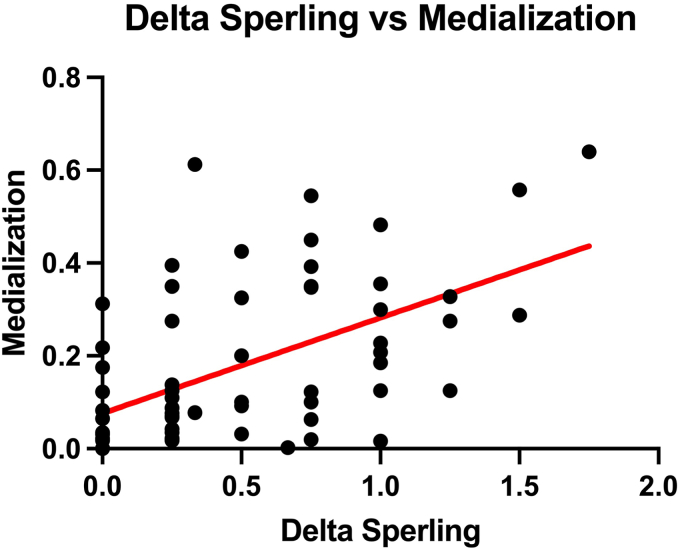
Moderate positive correlation between the delta of the Sperling classification and humeral medialization (r = 0.54; *P* < .0001; confidence interval = 0.33-0.70).

There was a moderate, positive correlation (r = 0.54; *P* < .0001; confidence interval = 0.33-0.70) between humeral medialization and increased (delta) glenoid erosion of the Sperling classification, defined as the difference between the Sperling grade at last follow-up and postoperatively (Fig. 5).

**Figure 6:**
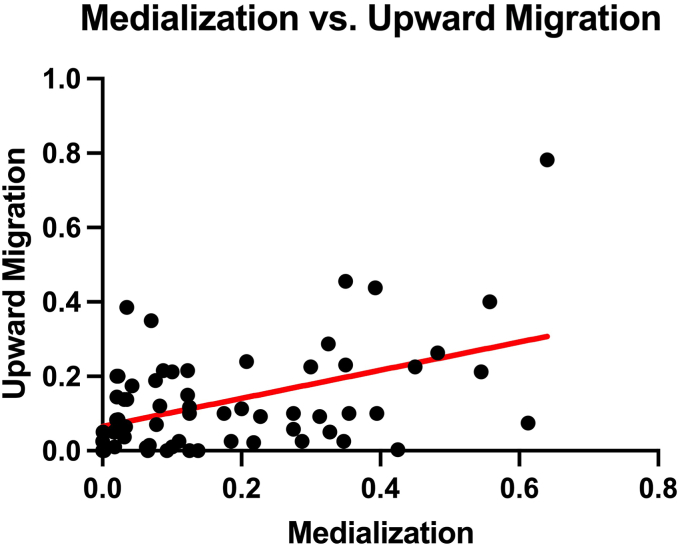
Weak positive correlation between humeral medialization and upward migration (r = 0.36; *P* = .003; confidence interval = 0.12–0.56).

Finally, there was a weak positive correlation between humeral upward migration and medialization (r = 0.36; *P* = .003; confidence interval = 0.12-0.56) (Fig. 6).

## Discussion

### Summary of results

The most important finding of the present study is that sequential standard AP radiographs in neutral rotation can be used to reliably measure humeral medialization and upward migration of the humeral head after HA implantation. The originality of our work is that we propose an easy-to-apply orthonormal frame on the AP radiographs. This orthonormal frame is defined by well-known and reliable landmarks: the supraspinatus-fossa line and the lateral aspect of the acromion. To avoid magnification errors, measurements are made in relation to the shaft diameter (d).

Of course, correctly angulated AP radiographs of the shoulder are required to apply our radiological method and make it reliable. The radiological method was validated by 4 observers on a relatively large dataset coming from different institutions. Furthermore, the moderate correlation between our measurement method for humeral medialization and the Sperling classification suggests that our quantitative measurement is superior to a qualitative scoring, like the Sperling classification.

Finally, another important finding of the present study is that HA-PyC, despite its low elastic modulus, still exhibits a certain degree of glenoid erosion, observed in about 60% of the patients. When comparing immediate postoperative and last AP radiographs taken after HA-PyC, we found that humeral medialization averaged 18% of the shaft diameters, while humeral upward migration averaged 13% of the shaft diameters. Interestingly, our method can even be used to assess the velocity of humeral medialization and migration after HA-PyC: we found that, when normalized per month, humeral medialization and upward migration averaged 0.57% and 0.44% of the shaft diameter, respectively.

### Reliability

The measurement reliability of the new method proved to be good to excellent (ICC = 0.88-0.96) and is thus comparable to those of established measurement methods.^23^ In contrast, the Sperling method, a qualitative assessment, achieved a slight reliability (Kappa = 0.16-0.19). While, to our best knowledge, the measurement reliability of the Sperling method has not yet been scientifically studied, other qualitative scorings of erosion or arthritis have shown equally disappointing results.^14^^,^^22^ In summary, this proves that a quantitative measurement can not only be more sensitive but also more reliable.

### Comparison to other measurement methods

Mercer et al proposed a measurement method to assess medialization using a template drawn upon the radiographs.^18^ Since their template is calibrated, they can use radiographs to measure metric distances. However, they tested their method only on 14 shoulders and did not perform a reliability study using ICCs. More importantly, their method only captures medialization, while our analysis shows that many implants undergo a cranially directed process of degeneration, which would be missed if implants were only followed on a horizontal axis. Other authors have tried similar methods but failed reproducing their measurements.^10^ Garret et al introduced a measurement method for pyrocarbon interposition shoulder arthroplasty using circles with increasing diameters starting at the center of the implant.^8^ However, they also did not validate their method with multiple observers and did not calculate ICCs.

### Does pyrocarbon hemiarthroplasty prevent glenoid erosion?

We found that the monthly rate of glenoid erosion was on average 0.57% of the shaft diameter. However, the relatively high standard deviation of 0.55% indicates that some patients exhibit negligible to no erosion, while others exhibit significant erosion even monthly. We analyzed frequency distributions (Fig. 3) and confirmed that about 39% of the patients showed almost no glenoid erosion. On the other hand, 61% of the patients exhibited glenoid erosion; in 31% of the patients, this glenoid erosion was higher than 1% of the shaft diameter. More research to identify risk factors for glenoid erosion despite the use of PyC is needed. Previous studies, although using the Sperling classification, suggest that humeral head oversizing might be an important risk factor.^4^ Our proposed measurement method can help precisely quantify the speed of medialization, thereby enabling the identification of risk factors in patients with pronounced medialization in future studies.

### Strengths and limitations

The main strength of the present study is that it introduces a reliable measurement method to quantitatively monitor humeral medialization and upward migration after HA implantation. Our new measurement method, based on standard true AP radiographs, proved to be reliable and immune to magnification errors. The main weakness of our method is the dependency on correctly angulated radiographs. It has been shown that different angulations or viewing perspectives on radiographs can produce diverging results.^5^^,^^9^^,^^12^^,^^25^ Thus, it is crucial to only use radiographs with correct angulation when applying the new measurements. Only true AP radiographs should be accepted, and they should comply with all the following parameters: (1) neutral rotation of the arm, (2) superimposition of the anterior and posterior glenoid rim, (3) visualization of glenohumeral space, and (4) foreshortening of the coracoid process but not of the scapular body. Finally, since the measurements from this study were not correlated to clinical results, the clinical significance is still unknown. However, this goes beyond the scope of a radiographic study.

## Conclusion

The new radiologic measurement method, based upon sequential postoperative AP radiographs, can be reliably used to measure humeral medialization and upward migration of HA over time. Despite a low elastic modulus, HA-PyC still leads to substantial glenoid erosion in about 60% of the patients. The here proposed measurement method can help precisely quantify the speed of medialization, thereby enabling the identification of risk factors in patients with pronounced medialization in future studies.

## Disclaimers:

Funding: No funding was disclosed by the authors.

Conflicts of interest: Pascal Boileau reports personal fees from Tornier/Stryker, outside the submitted work; and also has a patent Stryker with royalties paid.

Marc-Olivier Gauci reports personal fees from Stryker, outside the submitted work.

Any additional authors, their immediate families, and any research foundations with which they are affiliated have not received any financial payments or other benefits from any commercial entity related to the subject of this article.
